# Effects of Opioid Withdrawal on Psychobiology in People Living with HIV

**DOI:** 10.3390/v16010092

**Published:** 2024-01-06

**Authors:** Igor Grant, Evgeny Krupitsky, Marina Vetrova, Anya Umlauf, Robert K. Heaton, Richard L. Hauger, Olga Toussova, Donald R. Franklin, Scott L. Letendre, George Woody, Elena Blokhina, Dmitry Lioznov, Edwin Zvartau

**Affiliations:** 1 HIV Neurobehavioral Research Program, Department of Psychiatry, University of California San Diego, San Diego, CA 92103, USA; aumlauf@health.ucsd.edu (A.U.); rheaton@health.ucsd.edu (R.K.H.); rhauger@health.ucsd.edu (R.L.H.); dofranklin@health.ucsd.edu (D.R.F.); sletendre@ucsd.edu (S.L.L.); 2 Department of Pharmacology, Pavlov State Medical University, 197022 Saint Petersburg, Russia; kuenator@gmail.com (E.K.); mvetrova11@bk.ru (M.V.); otoussova@gmail.com (O.T.); blokhinaelena@gmail.com (E.B.); dlioznov@yandex.ru (D.L.); zvartau@gmail.com (E.Z.); 3 Department of Addictions, Bekhterev National Medical Research Center for Psychiatry and Neurology, 192019 Saint Petersburg, Russia; 4 Center for Behavior Genetics of Aging, University of California San Diego, La Jolla, CA 92093, USA; 5 Center of Excellence for Stress and Mental Health, VA San Diego Healthcare System, San Diego, CA 92093, USA; 6 Department of Medicine, University of California, San Diego, CA 92093, USA; 7 Department of Psychiatry, University of Pennsylvania, Philadelphia, PA 19104, USA; woodyg@pennmedicine.upenn.edu

**Keywords:** HIV, opioid, withdrawal, Russia

## Abstract

Objective: Many persons with opioid use disorders (OUDs) have HIV disease and experience clinically significant stress after they enroll in abstinence-based treatment and undergo medically assisted withdrawal. We examined whether opioid withdrawal affects virologic control, inflammatory markers, cognition, and mood in persons with an OUD and HIV, and explored whether measures of withdrawal stress, such as activation of the HPA axis, contribute to alterations in immune function, cognition, and mood. Method and participants: Study participants were 53 persons with HIV who were admitted for OUD treatment at the City Addiction Hospital in Saint Petersburg, Russian Federation. Participants were examined at admission, at the anticipated peak of withdrawal 3 to 7 days after the last day of a clonidine-based withdrawal process lasting 7 to 14 days, and 3 to 4 weeks after completing withdrawal. At these times, participants received medical exams and were evaluated for symptoms of withdrawal, as well as cognition and mood. Viral load, plasma cortisol, DHEA sulfate ester (DHEA-S), interleukin-6 (IL-6), and soluble CD14 (sCD14) were determined. Multivariable models examined the relationships between markers of HPA activation and the other parameters over time. Results: HPA activation as indexed by cortisol/DHEA-S ratio increased during withdrawal, as did markers of immune activation, IL-6 and sCD14. There were no significant associations between viral load and indicators of HPA activation. In longitudinal analyses, higher cortisol/DHEA sulfate was related to worse cognition overall, and more mood disturbance. Increase in IL-6 was associated with worse cognitive performance on a learning task. There were no significant associations with sCD14. Conclusions: Worsening of cognition and measures of mood disturbance during withdrawal were associated with activation of the HPA axis and some measures of inflammation. Whether repeated episodes of opioid withdrawal have a cumulative impact on long-term HIV outcomes and neurocognition is a topic for further investigation.

## 1. Introduction

Modern antiretroviral therapy (ART) does not fully protect from brain injury in people with HIV (PWH). As a result, neurocognitive impairment (NCI) remains a public health concern for the 34 million PWH in the world, particularly as they age. Psychoactive substance use (e.g., opioids) may contribute to this via several mechanisms. (1) The drugs can be neurotoxic, and this toxicity may interact with HIV effects on the brain; (2) the drugs and their dysregulation of opioid and dopamine pathways associated with addiction may alter neuroimmune function, which results in impaired virologic control, and/or worsen systemic inflammation. One mechanism that has received scant attention is that neuroimmune and hypothalamic–pituitary–adrenal (HPA) dysregulation during opioid withdrawal may set the stage for both acute, and possibly lasting, central nervous system (CNS) injury. Because people with opioid use disorder are likely to have repeated bouts of withdrawal during the course of their addiction, this repeated insult might have cumulative CNS effects.

Behavior associated with opioid addiction is a primary cause for HIV infection and transmission in Eastern Europe, including Russia [1]. In Russia, detoxification protocols do not involve opioid substitution and dose reduction, e.g., use of methadone. Rather, clonidine-assisted withdrawal, coupled with other supportive measures, is employed. As a result, some people experience significant withdrawal symptoms, and these can be accompanied by activation of the HPA axis, as well an immune dysregulation. Such HPA activation during opioid withdrawal could affect regulation of T lymphocytes, monocytes, myeloid cells, and macrophages, and worsen disruption of opioid-related gut and immune function. Such processes could facilitate microbial translocation [2,3,4] that can be linked to end organ damage and systemic inflammation favoring transport of HIV into tissue compartments, including the brain.

Neurosteroids, especially dehydroepiandrosterone (DHEA) and DHEA sulfate ester (DHEA-S), have beneficial actions on immune and brain systems [5,6,7], by exerting a “functional antagonism” of the cellular actions of glucocorticoids and thereby preventing detrimental effects of glucocorticoid receptor (GR) hypersignaling [8,9]. Furthermore, DHEA and DHEA-S help to counter-regulate the pathological effects of glucocorticoid hypersecretion and excessive allostatic load during high-stress states [6]. In the central nervous system, DHEA-S can improve memory retention while functionally antagonizing impairment of memory induced by glucocorticoids and N-methyl-D-aspartate (NMDA) or muscarinic receptor antagonists [5]. DHEA-S may enhance cognitive functioning, especially hippocampal-mediated memory processes, by allosterically modulating neuronal signaling at brain gamma-aminobutyric acid A (GABAA), NMDA, and sigma-1 receptors [5,6,10,11]. Furthermore, DHEA-S possesses a neurotrophic action by promoting dendritic arborization and survival of neocortical neurons [5,12]. Relevant to our current hypotheses and project, DHEA-S can prevent the development of opioid tolerance and dependence in preclinical animal models by modulating opioid, dopamine, glutamate, and GABA-A receptor signaling [5].

DHEA and DHEA-S also regulate cellular immune functions. DHEA-S can suppress the pro-inflammatory Th-2 cytokine pathway by inhibiting release of interleukins (e.g., IL-6) and tumor necrosis factor-alpha (TNFα) while downregulating cellular expression of COX-2 [9,13]. Additional anti-inflammatory actions of DHEA neurosteroids include the suppression of malondialdehyde (MDA, an indicator of lipid oxidation) and monocyte chemoattractant protein-1 (MCP-1) from endothelial cells [14]. During disease, CD4+ T-cells counts positively correlate with DHEA and DHEA-S and negatively correlate with plasma cortisol [15,16,17]. Furthermore, a low DHEA-S/cortisol ratio PWH is associated with higher HIV RNA in blood and a shift from Th1- to Th2-mediated immune responses [16]. This shift from Th1 cytokine production (facilitated by DHEA) to the Th2 pathway (promoted by glucocorticoids) may result in worse HIV clinical outcomes.

Protein expression of neurosteroid-synthesizing enzymes is reduced in human postmortem cerebral cortex from PWH [18]. This finding suggests that HIV disease suppresses the ability to synthesize and release neurosteroids in the CNS, in addition to markedly reducing synthesis and secretion of DHEA by the adrenal cortex. In vitro, DHEA-S inhibited release of IL-6 and TNF-α and HIV replication in monocyte-derived macrophages from PWH [18]. In a feline model of HIV disease, DHEA-S treatment was found to suppress pro-inflammatory gene transcripts (IL-6, TNF-α) and prevent neuron loss in cortex and basal ganglia, indicating DHEA-S may reduce neuroinflammation and neurodegeneration [18].

CD14 is a co-receptor with the Toll-like receptor, TLR4, that regulates immune function and inflammation [19]. In the periphery, CD14 is primarily expressed in monocytes and macrophages [19]. When immune cells shed CD14, it can be measured in plasma in its soluble form (sCD14). In the central nervous system, microglia express both CD14 and TLR4, while astrocytes express CD14 but not TLR4 [20]. Brain neurons do not express CD14 or TLR4 [20]. An important cellular mechanism for cognitive impairment and dementia involves the dysregulation of microglia, which promotes pathological neuroinflammation leading to neuronal cell death and neurodegeneration [21]. Importantly, microglia serve as a brain reservoir for latent HIV in ART-treated individuals with a suppressed viral load [22,23]. Furthermore, HIV-induced activation of microglia promotes neuroinflammation and the development of HIV-Associated Neurocognitive Disorder (HAND; [22,23]).

In our study, we measured circulating levels of cortisol and DHEA-S and calculated the cortisol/DHEA-S ratio as an index of HPA dysfunction. We chose to measure DHEA-sulfate rather than DHEA, since unsulfated DHEA is subject to diurnal changes while DHEA-S circulates in a steady state without variation. We determined whether opioid withdrawal-induced HPA and immune dysregulation as reflected by a rise in sCD14 and IL-6 acutely (during 7 days of detoxification) disrupts HIV control, and increases likelihood of poor cognitive performance and worsens mood. Additionally, we determined whether the extent of normalization of HPA axis biomarkers, and their relationship to sCD14 and IL-6 after a further 3 weeks of abstinence, were related to improved immune parameters, virologic control, and NC functioning.

## 2. Materials and Methods

### 2.1. Study Design

We conducted a longitudinal observational study to assess the pattern of the change in immune, hormonal, and cognitive function parameters during acute opioid withdrawal in 53 Russian, ART-naïve PWH with opioid use disorder, as well as one month after withdrawal. A total of 19% of participants used heroin, 38% used methadone (not prescribed), and 43% used both types of opioids. The primary opioid drug was based on urine drug screen.

### 2.2. Study Setting

Between February 2015 and July 2016, study participants were recruited from inpatient detoxification wards of the City Addiction Hospital (CAH) located in Saint Petersburg, Russia. The hospital, which is government-funded and has a capacity of 500 beds, offers free addiction care to the residents of St. Petersburg who register as patients with substance use disorder (e.g., alcohol or drug). Services provided include detoxification (e.g., withdrawal medical treatment), early stabilization, treatment of psychiatric and somatic disorders, and in- and outpatient long-term rehabilitation. The usual length of stay for patients admitted to the hospital ranges from one to three weeks, including 7–14 days of detoxification treatment, depending on the type of opioids used. Clonidine, antidepressants, non-opioid analgesics, hypnotics, and loperamide are the most common drugs used to treat opioid withdrawal in Russia.

### 2.3. Participants

The study enrolled 53 participants who met the following inclusion criteria: (1) age 18 years or older; (2) diagnosis of HIV infection; (3) no prior or current ART at the time of inclusion; (4) diagnosis of opioid dependence according to ICD-10 criteria; (5) ability to read, understand the purpose of the study, and provide informed consent form; and (6) willingness and ability to follow the protocol procedures. Exclusion criteria for the study enrollment included (1) advanced HIV disease requiring specialized medical treatment; (2) currently psychotic as determined by psychiatric examination (schizophrenia, paranoid disorder, mania); and (3) suicidal or homicidal ideation requiring immediate attention.

All patients admitted to the detoxification department of the CAH who had positive HIV status (which is routinely obtained during the admission process) were eligible for screening, which was conducted by Research Assessors (who were trained CAH physicians (narcologists) working in the detoxification department). Once a patient was identified as eligible for enrollment into the study, participation in the study was offered, and the informed consent was administered and documented. This study was approved by Institutional Review Boards of the University of California, San Diego, and the corresponding IRB at the First St. Petersburg Pavlov State Medical University.

### 2.4. Study Assessments

Participants in our study were assessed at four visit points over the withdrawal treatment and after its completion: T1 (baseline)—the first day of hospitalization (start of detoxification); T2—the anticipated peak of the withdrawal (at 3–7 days after T1); T3—the last day of detoxification (7–14 days after T1); T4—the last day of stabilization (at 3–4 weeks after T1). The assessment protocol consisted of (1) face-to-face structured interview at T1, T3, and T4 study visits; (2) daily assessment of withdrawal symptom severity (Clinical Opiate Withdrawal Scale (COWS)) and mood (Profile of Mood States (POMS)) from T1 to T3 and once at T4; (3) neurocognitive assessment at T1, T3, and T4; (4) blood draw (T2, T3, and T4); (5) urine drug test (T1, T3, and T4). These data were supplemented by reviewing medical records for routinely obtained laboratory measures including complete blood count (CBC), chemistry panel (including liver enzymes (ALT and AST)), and HCV and syphilis serology.

Between T1 and T2, the data about baseline patient characteristics were collected and included the following parameters: demographic characteristics, behavioral notes, HIV Risk Assessment Battery [24], Modified Neurobehavioral Medical Screen (surveys medical and developmental history for non-HIV conditions that are likely to be associated with neurobehavioral compromise, e.g., learning disability, serious head injury, seizure disorder), lifetime and current substance use history (including self-reported estimate of number of symptomatic withdrawal episodes), psychiatric assessment (via the Mini International Neuropsychiatric Interview (MINI: structured diagnostic interview for DSM-IV and ICD-10 psychiatric disorders)), brief neuromedical history (focused on current or past non-HIV medical conditions), HIV diagnosis characteristics (via CDC Classification Worksheet and Diagnosis ICD-9 Worksheet). At the T4 visit, the assessments were updated to include substance use history, brief neuromedical history, and a medication summary which included information about the name of prescribed medications, doses, and dates of taking medications.

### 2.5. Neurocognitive Assessment

The neurocognitive battery was selected to provide a brief (approximately 30 min) assessment of the key cognitive domains that are frequently compromised in HIV disease and drug use disorders because of known predilection to frontostriatal injury (Table 1). Tests were translated into Russian and administered by CAH staff (clinical psychologists) who were trained by the US team.

### 2.6. Mood and Withdrawal Assessments

Multiple dimensions of current mood were assessed by self-report using the Russian language version of the POMS [33], which provides a total mood disturbance score and data on 6 mood dimensions, including tension/anxiety, fatigue/inertia, confusion/bewilderment, vigor/activation, anger/hostility, and dejection/depression. To assess common symptoms and signs of opioid withdrawal, we employed the clinician-rated COWS [34], which provides ratings on 11 dimensions, as well as a summary score.

### 2.7. Neuroendocrine Immunoassay Methods

Plasma concentrations of cortisol were measured using an enzyme-linked immunosorbent assay (ELISA) that has a working range up to 2000 nmol/L, sensitivity of 10 nmol/L, and intra-assay CV of 8% (Alkor Bio. St. Petersburg, Russia). Plasma concentrations of DHEA-S were measured using an ELISA that has a working range up to 10 IU/mL, sensitivity of 0.04 IU/mL, and intra-assay CV of 8% (Alkor Bio St. Petersburg, Russia). Immunoassays were performed at the Laboratory of Molecular Immunology and Seroepidemiology at the St. Petersburg Pasteur Institute.

### 2.8. HIV RNA and Immune Activation Methods

HIV-associated immune activation and dysregulation involves T-cells, monocyte/macrophages, B-cells, and other components of the immune system. Many approaches to describing the immune abnormalities of HIV disease have been evaluated, e.g., measurement of cellular and soluble factors in blood and other fluids. Considering the limited scale of this project, we focused on a marker of monocyte/macrophage activation (soluble CD14), as well as an activation-associated soluble biomarker (IL-6). Soluble CD14 is the soluble form of a bacterial lipopolysaccharide receptor that reflects microbial translocation resulting from HIV-damaged gut-associated lymphoid tissue and possibly opioid-induced gut mucosal injury [2,3,4]. Though this panel has limitations, similar panels have been linked to transient viremia during ART [35], CD4+ T-cell loss [36], vascular disease [37,38], neurocognitive impairment [2], and low functional status during ART [39]. We stored specimens to enable future analyses of additional cellular and soluble biomarkers.

HIV RNA was measured by real-time polymerase chain reaction on a validated, automated system with a lower limit of quantitation of 40 copies/mL (Abbott Molecular, Des Plaines, IL, USA). T cell subsets were analyzed in whole blood using 4-color flow cytometry and were defined as activated CD4+ cells (HLA-DR+) or activated CD8+ cells (HLA-DR+, CD38+). Soluble biomarkers were measured by commercial immunoassays. For example, plasma concentrations of IL-6 were measured with a high-sensitivity assay with sensitivity of 0.04 pg/mL, an intra-assay CV of 7.4 ± 0.2%, and an intra-assay CV of 8.0 ± 0.8% (R&D Systems, Minneapolis, MN, USA).

### 2.9. Statistical Analyses

A significant batch effect was observed for the biomarkers, IL-6 and sCD14. For this reason, their values were transformed, first by taking log_10_ to normalize their distribution and then by standardizing log_10_-transformed values within each batch, using means and standard deviations (SDs) from the baseline measurement. Hereafter, the notation _z_log_10_ is used to indicate the transformed values. In addition to analyzing cortisol and DHEA-S levels independently, we also analyzed the cortisol/DHEA-S ratio, which has been established to provide an index of “functional antagonism” of cortisol action by DHEA [8,9]. Values of cortisol/DHEA-S ratio were log_10_ transformed.

Mixed-effects models with subject-specific random intercepts were used for data measured longitudinally to account for within-subject correlation. Model specifications were chosen based on the lowest AIC value associated with the best model fit. Study visit (visits T1, T3, T4) was included in all models as a fixed effect. The first set of models examined change in cognitive and behavioral outcomes over three visits. The results are presented as Cohen’s d and its 95% confidence interval (CI_95_), calculated by dividing model coefficient estimates by residual standard deviation. Additional models examined the association between cognitive and behavioral outcomes with biomarkers. Effect size is reported as coefficient (coef) and its standard error (SE) representing change in the outcome per one unit increase in the predictor, and as partial R-squared [40]. Models involving biomarkers included biomarker batch (batch 1, 2) as fixed effects. Additionally, models for association between markers and cognitive and behavioral outcomes controlled for participant’s age, sex, education, HIV viral load (>500, ≤500; time-varying), and psychotropic medications. In these models, the markers were treated as time-varying predictors. For related outcomes, such as scaled scores for individual cognitive tests, mean scaled scores for cognitive domains, and POMS subscores, two *p*-values are shown: unadjusted (*p*) and adjusted (adj. *p*) for multiple testing using the false discovery rate (FDR) method. Bivariate associations between 2 numeric measures were assessed with Pearson’s correlation test.

Analyses were performed using R v4.2.1 [41] and the package *nlme* [42]. All tests were two-sided with a significance level of 0.05.

## 3. Results

### 3.1. Demographics

The study cohort consisted of 53 participants, on average 34.7 (SD = 4.5) years old, 88.7% men, with an average education of 11.3 years (SD = 1.9). At the time of enrollment, all participants were HIV-positive and ART-naïve with uncontrolled viral load (median = 4.38, range = 2.7–4.98 log_10_ copies/mL) and low CD4 count (mean = 272 cells/mm^3^, range = 40–654, N = 32 with available data for CD4 count), 96.1% of whom had hepatitis C coinfection (N = 51). Additional cohort characteristics are listed in Table 2.

### 3.2. Cognitive and Behavioral Changes over Time

**Figure 1:**
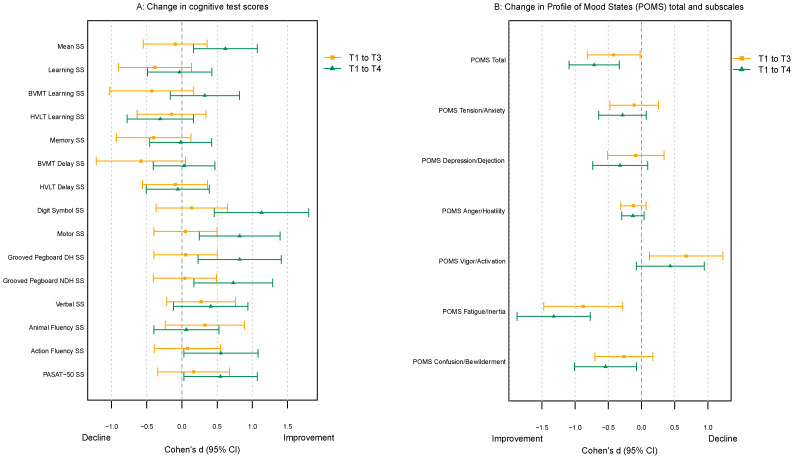
Mixed-effects models with subject-specific random effects were used to model change in (**A**) cognitive and (**B**) emotional/behavioral outcomes over three visits. The results are presented in the form of Cohen’s d and its 95% confidence interval (CI_95_), calculated by dividing model coefficient estimates by residual standard deviation. Change is displayed from baseline to T3 and baseline to T4. In (**A**), SS refers to scaled scores, and “mean” refers to overall cognitive score from averaging all cognitive test scale scores. (**B**) displays change in POMS total mood disturbance and component scales.

Visits T3 and T4 took place at a median 10 (range 4–15) and 27 (19–84) days from baseline. Although little change occurred on the mean cognitive scaled scores from T1 to T3 (contrary to normal expectations for improvements due to “practice effects”), there was a significant improvement in cognitive global mean scaled score by T4 (d = 0.62; CI_95_ 0.16, 1.07; *p* = 0.008); also, whereas learning and memory performances actually dropped somewhat at T2, they recovered by T3 (see Figure 1). On a test level, again only at T4, improvements were observed for the following tests: Digit Symbol (d = 1.13; CI_95_ 0.46, 1.80; adj. *p* < 0.001), Grooved Pegboard Dominant Hand (d = 0.82; CI_95_ 0.23, 1.41; adj. *p* = 0.002) and Non-dominant Hand (d = 0.73; CI_95_ 0.17, 1.29; adj. *p* = 0.006), Action Fluency (d = 0.55; CI_95_ 0.03, 1.08; adj. *p* = 0.037), and PASAT-50 (d = 0.55; CI_95_ 0.03, 1.07; adj. *p* = 0.037). Figure 1A summarizes the results of these analyses.

The POMS mood/behavioral scores exhibited more immediate improvement (Figure 1B). Specifically, POMS total score was significantly reduced by visit T3 (d = −0.42; CI_95_ −0.82, −0.02; *p* = 0.040) and further improved by visit T4 (d = −0.71; aCI_95_ −1.09, −0.33; adj. *p* < 0.001). A similar pattern was observed for POMS fatigue/inertia with improvements at visit T3 (d = −0.88; CI_95_ −1.47, −0.28; adj. *p* = 0.001) and visit T4 (d = −1.32; aCI_95_ −1.87, −0.77; adj. *p* < 0.001). POMS confusion/bewilderment score did not show a significant improvement at T3 (adj. *p* = 0.292), but its average score was significantly lower at visit 4 (d = −0.54; CI_95_ −1.01, −0.07; adj. *p* = 0.017). POMS vigor/activation score increased significantly at visit 2 (d = 0.67; CI_95_ 0.12, 1.23; adj. *p* = 0.011), but at T4, its average score did not differ statistically from baseline (d = 0.43; CI_95_ −0.08, 0.95; adj. *p* = 0.114). These patterns were also observed in multivariable models testing for association between POMS scores and biomarkers (results described below).

### 3.3. Medication Use Associations with Cortisol/DHEA-S, IL-6, and sCD14

Medication information was available for 47 participants, of whom 38 received one or more of the following: 21 clonidine, 8 chlorprothixene, 32 droperidol. Results of tests for associations between markers and medication use are shown in Table 3. Participants with higher log_10_ cortisol/DHEA-S ratio were more likely to be on clonidine (*p* = 0.002) and droperidol (*p* = 0.043). Droperidol use was associated with higher levels of _z_log_10_ sCD14 (*p* = 0.003). Average levels of _z_log_10_ IL-6 were higher for those receiving clonidine and droperidol users, but neither association reached statistical significance (*ps* = 0.154 and 0.108, respectively).

### 3.4. Association between Cortisol/DHEA-S and IL-6, sCD14

A multivariable model, controlling for viral load level (<=500, >500), showed a positive but not statistically significant association between log_10_ cortisol/DHEA-S and _z_log_10_ IL-6 (coef = 0.435, SE = 0.232, *p* = 0.064). No consistent results were found when testing for an association between changes in log_10_ cortisol/DHEA-S and changes in _z_log_10_ IL-6, where changes were calculated as differences between values measured at visits T2 and T3, and visits T3 and T4.

A multivariable model, controlling for viral load level (<=500, >500) and visit, showed a negative and significant association between log_10_ cortisol/DHEA-S and _z_log_10_ sCD14 (coef = −0.561, SE = 0.211, *p* = 0.009). Pearson’s correlation test suggests that an increase in _z_log_10_ sCD14 was associated with a decrease in log_10_ cortisol/DHEA-S from visit T2 to visit T3 (r = −0.26, *p* = 0.065) and from visit T3 to visit T4 (r = −0.30, *p* = 0.034).

### 3.5. Association between Cortisol/DHEA-S and Cognition

Multivariable longitudinal models showed that lower (worse) cognitive scaled scores were consistently associated with higher levels of log_10_ cortisol/DHEA-S (Table 4). On average, an increase in 1 log_10_ units of cortisol/DHEA-S was associated with a global mean scaled score decrease by 1.11 points (*p* = 0.020). Among individual cognitive tests, many showed a similar trend, with coefficients ranging from −0.97 to −1.28, but did not reach statistical significance, including Grooved Pegboard for dominant hand (*p* = 0.132), BVMT-R learning (*p* = 0.057), HVLT-R delayed recall (*p* = 0.119), Digit Symbol (*p* = 0.086), and PASAT-50 (*p* = 0.163). Only one individual test showed statistically significant association: Grooved Pegboard for non-dominant hand (coef = −2.38, SE = 0.82, *p* = 0.005). The FDR-adjusted p-value for this test remained significant (*p* = 0.049). Models for domain scaled scores showed a statistically significant negative association for the motor domain (coef = −1.82, SE = 0.80, *p* = 0.025) but non-significant negative correlations with learning scaled score (coef = −0.816, SE = 0.55, *p* = 0.139), memory scaled score (coef = −1.04, SE = 0.66, *p* = 0.116), and verbal scaled score (coef = −0.31, SE = 0.53, *p* = 0.558). None of the domain associations remained statistically significant after adjustment for multiple testing (*ps* > 0.100).

### 3.6. Association between IL-6 and Cognition

The association between _z_log_10_ IL-6 and the cognitive global mean scaled scores was negative but not statistically significant (coef = −0.15, SE = 0.11, *p* = 0.178). Among individual cognitive tests, two significant associations were found: HVLT-R learning (coef = −0.43, SE = 0.17, *p* = 0.014) and Digit Symbol (coef = −0.26, SE = 0.12, *p* = 0.042), but with FDR-adjusted p-values not reaching significance (0.139 and 0.208, respectively). Learning (coef = −0.30, SE = 0.13, *p* = 0.017, adj. *p* = 0.070) domain scaled scores showed the strongest negative association with this biomarker. Negative but not significant associations were also observed for the memory domain scaled scores (coef = −0.23, SE = 0.15, *p* = 0.133). Full results are shown in Table 4.

### 3.7. Association between sCD14 and Cognition

There were no significant associations between cognitive test scores and plasma sCD14 concentrations (Table 4).

### 3.8. Association between Cortisol/DHEA-S, IL-6, sCD14 and POMS

In the multivariable model adjusting for covariates, the association between log_10_ cortisol/DHEA-S and POMS total score was positive, but not statistically significant (coef = 9.89, SE = 8.74, *p* = 0.261); a positive association with POMS total scores means that high levels of the biomarker were associated with worse affective status. Positive and significant associations were detected for log_10_ cortisol/DHEA-S and two POMS subscales: depression/dejection (coef = 6.03, SE = 2.81, *p* = 0.035) and anger/hostility (coef = 3.61, SE = 1.18, *p* = 0.003) (Table 5). The FDR-adjusted p-value was significant for the second subscale only: depression/dejection (adj. *p* = 0.105) and anger/hostility (adj. *p* = 0.018). Multivariable models revealed no significant relationships between POMS variables and _z_log_10_ IL-6 nor _z_log_10_ sCD14, with the exception that greater confusion/bewilderment related to lower _z_log_10_ sCD14, which was opposite to prediction, and might possibly reflect a complex interaction among log_10_ cortisol/DHEA-S, _z_log_10_ sCD14, and the POMS variable, which we did not have the power to test, or a chance result (Table 5).

## 4. Discussion

Our study suggests that withdrawal in individuals with opioid dependence who are also HIV-infected dysregulates the HPA axis, negatively affects cognitive function, induces dysphoric symptoms, and is associated with a rise in the inflammatory marker IL-6. More specifically, we noted that in longitudinal models taking into account data from repeated measures throughout the first month of abstinence, a higher ratio of cortisol to DHEA-S was associated with worse performance on a global index of cognition, with global mean scaled score decreasing by 1.11 points for 1 log_10_ unit increase in the cortisol/DHEA-S ratio. Higher cortisol/DHEA-S ratio was also related to more dysphoric symptoms, such as anger/hostility and depression/dejection.

Many studies in people without HIV infection have established that excessive cortisol secretion and glucocorticoid receptor hypersignaling contribute to cognitive impairment, including the development of Alzheimer’s disease [43]. However, cortisol is only one of several physiologically active neurosteroids, and there is evidence that their interaction can have complex regulatory actions. Specifically, dehydroepiandrosterone (DHEA) and the sulfate ester DHEA-S are neurosteroids that have beneficial actions on immune and brain function [5,6,7,8,9]. Furthermore, DHEA and DHEA-S have been shown to exert a “functional antagonism” of the cellular actions of cortisol by dampening its detrimental effects of excessive glucocorticoid receptor signaling, which results in high allostatic load and stress pathophysiology [6,8,9]. This interaction is important for immune function and HIV outcomes, considering that cortisol has an immunosuppressive action, while DHEA has an immunostimulatory action [15,16,17]. In the central nervous system, DHEA-S has been found to enhance hippocampal-mediated memory processes and to have neurotrophic and neuroprotective actions, while cortisol has the opposite effects [5,6,12,43]. Such effects could contribute to HIV-associated neurocognitive disorder (HAND).

Therefore, we chose to investigate HPA dysregulation and its possible detrimental impact on PWH during opioid withdrawal by analyzing the cortisol/DHEA-S ratio, since this parameter provides a useful measure of the relative activity of both adrenocortical steroids and an index of HPA dysfunction. Consistent with our findings relating worse global cognition (including learning, which is a key aspect of episodic memory) and more dysphoric symptoms to higher cortisol/DHEA-S ratio, prior studies have reported that elevated cortisol/DHEA-S ratios were associated with memory impairment as well as depression and high stress states [44,45,46].

We found a highly significant, negative association between log_10_ cortisol/DHEA-S ratio and log_10_sCD14 (r = 0.561, p = 0.009). Alterations in the cortisol/DHEA-S ratio can result from normal or increased levels of cortisol or from lower levels of DHEA-S. In our study, we observed a dramatic elevation of cortisol levels during opioid withdrawal in the presence of low DHEA-S levels. Glucocorticoid receptors (GRs) are highly expressed in macrophages and monocytes in the periphery, including myeloid tissue in the small intestine and colon [47]. Cortisol stimulation of GR signaling in the intestinal epithelium controls macrophages and monocytes, regulating tissue homeostasis and preventing excessive inflammation under normal conditions [47]. In contrast, pro-inflammatory pathways are pathologically upregulated in transgenic mice with a knockout of the GR gene that eliminates glucocorticoid-mediated tissue homeostasis [47]. Glucocorticoids reduce the expression of membrane CD14 in monocytes and decrease circulating levels of sCD14 [48], which is consistent with our data showing an inverse relationship between log_10_ cortisol/DHEA-S ratio and log_10_sCD14 during opiate withdrawal. Although glucocorticoids negatively regulate IL-6 by inhibiting the promoter on the IL-6 gene and suppressing IL-6 levels [49], we found no significant relationship between the log_10_ cortisol/DHEA-S ratio and log_10_ IL-6.

While our findings are consistent with the notion that opioid withdrawal-induced HPA dysregulation may have several negative consequences, there are several limitations to our study. The sample size is relatively modest, heightening the possibility of false discovery. At the same time, the study did benefit from the added precision of a repeated-measures design, and we took steps to appropriately adjust analyses for a number of covariates and possible confounders. Because ours was a clinical population, participants received a number of medications, most notably clonidine, that can have effects on HPA and immune functioning. Our participants were ART-naïve, and that limits generalization to PWH with opioid dependence who are receiving ART. At the same time, in many global contexts, persons dependent on opioids are only discovered to be HIV-infected incidental to their addiction treatment, and may undergo withdrawal before they can access ART.

In summary, we noted that HPA dysregulation attendant on opioid withdrawal was related to cognitive and mood disturbance, and also alterations in markers of immune activation in a group of PWH. We found no effect on viral load in PWH not receiving ART. If our observations are confirmed, future studies may need to model histories of repeated withdrawal episodes as additional mechanisms into understanding the persistence of neurocognitive impairment in PWH in the era of modern HIV care.

## Figures and Tables

**Table 1:** Neurocognitive assessment battery.

Cognitive Domain	Test
Attention/Speed of Information Processing	Digit Symbol/Coding subtest from Wechsler Adult Intelligence Scale-III (WAIS-III) [25] Color Trails Test 1 [26] Paced Auditory Serial Addition Test (PASAT-50) [27]
Memory (learning and recall of verbal and nonverbal/visual information)	Hopkins Verbal Learning Test—Revised [28] Brief Visuospatial Memory Test—Revised [29]
Executive Functioning	Color Trails Test 2 [26]
Verbal Fluency	Category Fluency (Animals) [30] Action Fluency [31]
Motor speed and coordination	Grooved Pegboard Test (Dominant and Non-dominant) [32]

**Table 2:** Study cohort description at baseline (N = 53).

Variable	Mean (SD) or N (%)	Range	N (%) Missing
Age (years)	34.7 (4.45)	23.0–46.0	0 (0%)
Female sex	6 (11.3%)		0 (0%)
Education (years)	11.3 (1.91)	7.0–17.0	0 (0%)
Log_10_ HIV VL	4.38 [4.08, 4.61]	2.70–4.98	0 (0%)
HIV VL *≤* 500	12 (22.6%)		0 (0%)
CD4 Count	272 (154)	40–654	21 (39.6%)
HBV	1 (1.9%)		1 (1.9%)
HCV	49 (96.1%)		2 (3.8%)
Syphilis	1 (1.9%)		1 (1.9%)
WBC	6.00 [4.50, 7.69]	2.90–49.00	2 (3.8%)
RBC	4.60 [4.34, 5.00]	3.26–14.30	2 (3.8%)
HGB	140 [132, 153]	17–167	2 (3.8%)
PLT	173 [143, 249]	14–350	3 (5.7%)
LYM	31.1 [19.5, 41.3]	1.2–56.5	3 (5.7%)
MON	4.62 [3.48, 5.90]	0.32–378.00	9 (17%)
RDW	12.3 [11.2, 13.7]	4.8–47.2	5 (9.4%)
MPV	7.70 [6.50, 8.94]	4.30–19.90	6 (11.3%)
Total Protein	77.1 [71.1, 82.2]	55.9–103.7	3 (5.7%)
Total Bilirubin	8.40 [5.53, 13.70]	1.80–55.90	3 (5.7%)
Direct Bilirubin	6.25 [3.17, 8.65]	1.10–10.60	47 (88.7%)
Cholesterol	3.30 [2.90, 4.10]	2.10–4.70	24 (45.3%)
AST	43.7 [24.5, 64.5]	4.3–309.0	2 (3.8%)
ALT	37.8 [21.5, 61.1]	5.7–160.0	3 (5.7%)
Glucose	5.04 [4.48, 5.50]	2.82–7.30	5 (9.4%)
Marijuana Utox+	0 (0%)		0 (0%)
Cocaine Utox+	0 (0%)		0 (0%)
Morphine Utox+	40 (75.5%)		0 (0%)
Methadone Utox+	40 (75.5%)		0 (0%)
Methamphetamine Utox+	0 (0%)		0 (0%)
Amphetamine Utox+	0 (0%)		0 (0%)

HBV = hepatitis B virus; HCV = hepatitis C virus; WBC = white blood cell; RBC = red blood cell; HGB = hemoglobin; PLT = platelet; LYM = lymphocyte; MON = monocyte; RDW = red cell distribution width; MPV = mean platelet volume; AST = aspartate aminotransferase; ALT = alanine transaminase; Utox = urine drug screen.

**Table 3:** Association of cortisol measures and biomarkers with medication use (N = 47).

	Log_10_ Cortisol/DHEA		Cortisol		_z_log_10_ IL-6 ^#^		_z_log_10_ sCD14 ^#^	
Medication Use	Coef. (SE)	*p*	Coef. (SE)	*p*	Coef. (SE)	*p*	Coef. (SE)	*p*
Clonidine	**0.237 (0.073)**	**0.002**	**222 (68)**	**0.002**	**0.512 (0.353)**	**0.154**	0.043 (0.306)	0.890
Chlorprothixene	0.099 (0.106)	0.354	−3 (100)	0.973	−0.277 (0.323)	0.396	−0.187 (0.306)	0.545
Droperidol	**0.171 (0.082)**	**0.043**	155 (77)	0.051	0.458 (0.279)	0.108	**0.771 (0.243)**	**0.003**
Antipsychotics	**0.207 (0.097)**	**0.039**	106 (94)	0.263	0.383 (0.309)	0.221	**0.782 (0.273)**	**0.006**

^#^ Controlled for batch (2 batches). Participants could be prescribed more than one medication.

**Table 4:** Association of log_10_ cortisol/DHEA, _z_log_10_ IL-6, and _z_log_10_ sCD14 with cognitive outcomes, as estimated by three separate multivariable models.

	log_10_ Cortisol/DHEA			_z_log_10_ IL-6			_z_log_10_ sCD14		
Outcome (Scaled Score)	Coefficient (SE)	*p*	adj. *p*	Coefficient (SE)	*p*	adj. *p*	Coefficient (SE)	*p*	adj. *p*
Global Mean	**−1.110 (0.470)**	**0.020**	--	−0.147 (0.108)	0.178	--	0.335 (0.173)	0.057	--
Learning Domain	−0.816 (0.547)	0.139	0.185	**−0.303 (0.125)**	**0.017**	0.070	0.178 (0.203)	0.381	0.381
BVMT-R Learning	−1.234 (0.639)	0.057	0.264	−0.177 (0.155)	0.256	0.565	0.316 (0.241)	0.193	0.465
HVLT-R Learning	−0.645 (0.745)	0.389	0.486	**−0.429 (0.171)**	**0.014**	0.139	−0.039 (0.272)	0.885	0.885
Memory Domain	−1.039 (0.655)	0.116	0.185	−0.234 (0.154)	0.133	0.266	0.272 (0.234)	0.249	0.332
BVMT-R Delayed Recall	−0.876 (0.765)	0.255	0.364	−0.175 (0.179)	0.332	0.565	0.364 (0.268)	0.178	0.465
HVLT-R Delayed Recall	−1.249 (0.793)	0.119	0.264	−0.293 (0.191)	0.128	0.428	0.154 (0.288)	0.593	0.709
Motor Domain	**−1.822 (0.799)**	**0.025**	0.100	−0.060 (0.186)	0.748	0.748	0.434 (0.295)	0.145	0.332
Grooved Pegboard—Dominant Hand	−1.279 (0.841)	0.132	0.264	0.030 (0.196)	0.878	0.964	0.339 (0.308)	0.273	0.465
Grooved Pegboard—Non-Dominant Hand	**−2.375 (0.823)**	**0.005**	**0.049**	−0.151 (0.193)	0.437	0.565	0.516 (0.307)	0.097	0.465
Verbal Domain	−0.310 (0.528)	0.558	0.558	0.067 (0.117)	0.570	0.748	0.252 (0.198)	0.206	0.332
Animal Fluency	−0.418 (0.664)	0.531	0.545	0.007 (0.156)	0.964	0.964	0.275 (0.253)	0.279	0.465
Action Fluency	−0.433 (0.712)	0.545	0.545	0.119 (0.157)	0.452	0.565	0.153 (0.263)	0.562	0.709
WAIS-III Digit Symbol *	−0.966 (0.557)	0.086	0.264	**−0.256 (0.124)**	**0.042**	0.208	**0.652 (0.228)**	**0.005**	0.053
PASAT-50	−1.147 (0.815)	0.163	0.272	−0.160 (0.190)	0.403	0.565	0.142 (0.301)	0.638	0.709

Models controlled for biomarker batch (2 batches, IL-6 and sCD14 models only), participant’s age, sex, education, HIV viral load (>500, <=500), antipsychotics use, and visit (3 visits). Domain scaled scores are average values of individual test scaled scores. N = 47, * N = 46. P is unadjusted *p*-value; adj. *p* is *p*-value FDR-adjusted for multiple testing. Coefficient and standard error (SE) represent change in cognitive scaled score per one unit increase in biomarker, adjusted for covariates.

**Table 5:** Association of log_10_ cortisol/DHEA, _z_log_10_ IL-6, and _z_log_10_ sCD14 with POMS outcomes, as estimated by three separate multivariable models.

	log_10_ Cortisol/DHEA			_z_log_10_ IL-6			_z_log_10_ sCD14		
POMS Scores	Coefficient (SE)	*p*	adj. *p*	Coefficient (SE)	*p*	adj. *p*	Coefficient (SE)	*p*	adj. *p*
Total Score	9.86 (8.74)	0.261	--	1.76 (1.73)	0.314	--	−4.87 (2.81)	0.086	--
Tension/Anxiety	−1.32 (1.56)	0.400	0.480	−0.26 (0.36)	0.480	0.697	−0.67 (0.55)	0.230	0.460
Depression/Dejection	**6.03 (2.81)**	**0.035**	0.105	0.72 (0.58)	0.219	0.656	−0.83 (1.10)	0.455	0.683
Anger/Hostility	**3.61 (1.18)**	**0.003**	**0.018**	0.37 (0.22)	0.105	0.631	−0.07 (0.45)	0.886	0.886
Vigor/Activation	−1.42 (1.50)	0.347	0.480	−0.20 (0.36)	0.581	0.697	0.29 (0.56)	0.607	0.728
Fatigue/Inertia	0.25 (1.58)	0.873	0.873	0.06 (0.37)	0.876	0.876	−0.89 (0.56)	0.117	0.350
Confusion/Bewilderment	1.99 (1.22)	0.107	0.213	0.24 (0.26)	0.356	0.697	**−1.01 (0.42)**	**0.018**	0.111

Models controlled for biomarker batch (2 batches, IL-6 and sCD14 models only), participant’s age, sex, education, HIV viral load (>500, <=500), antipsychotics use, and visit (3 visits). *p* is unadjusted *p*-value; adj. *p* is *p*-value FDR-adjusted for multiple testing. Coefficient and standard error (SE) represent change in POMS score per one unit increase in biomarker, adjusted for covariates.

## Data Availability

Data are available through the HIV Neurobehavioral Research Program and the University of California, San Diego (hnrpresource@ucsd.edu).
